# Use of Pseudoephedrine in the Management of Severe Clozapine Induced Urinary Urgency and Frequency

**DOI:** 10.1192/bjo.2022.369

**Published:** 2022-06-20

**Authors:** Tongeji E Tungaraza

**Affiliations:** Priory Healthcare, Birmingham, United Kingdom

## Abstract

**Aims:**

Clozapine has several effects on the genitourinary system including enuresis and an overactive bladder (OAB). However, little if any has been written about its effect in causing OAB and its prevalence. Typical feature of OAB is urinary urgency associated with urinary frequency in the absence of urinary tract infection or other obvious pathology. Having 8 voids or more in 24 hours in addition to urinary urgency is strongly suggestive of OAB. AB developed AOB while being treated with clozapine.

**Methods:**

AB is 33 years old white lady with a well-established diagnosis of emotionally unstable personality disorder. She has been in mental health services from her late teens. She has had a trial of several antipsychotics, both oral and depot and has had a number of DBT sessions and one EMDR with limited effect. Prior to this episode, AB has had two successful trials of clozapine, one resulting in her longest period (9 months) of living in the community in 1998.

AB was started on clozapine again in 2021, she developed urinary frequency. She requested it to be stopped. Her condition deteriorated such that she needed intensive care unit. AB was again started on clozapine. She developed urinary frequency once more when the dose reached 250 mg. She did not have infection. For one week, her urinary frequency ranged from 36–66 in 24 hours. It was more evident in the day time. There was no incontinence. AB remained in her room close to her toilet. She stopped engaging in her therapy, she did not mix with others and did go out. She tried a number of medications-aripiprazole, oxybutynin and desmopressin with no benefit. Pseudoephedrine was started at 15 mg twice a day, eventually reaching 30 mg three times a day. Within two weeks, the urge disappeared and the frequency normalised. Six months on, AB remains well and has not reported any side effects.

**Results:**

Pseudoephedrine, an indirect alpha-adrenergic agonist successfully normalised AB's urinary frequency. It was evident so within few days though the maximum benefit was noted within two weeks. This is in agreement with what is reported in cases with enuresis

**Conclusion:**

Very little is known about clozapine induced OAB. It had a severe negative impact on AB, who failed to engage in her therapy and her social life. Pseudoephedrine brought relief within a short period of time. In case of OAB and when stopping clozapine is not an option because of underlying severe mental disorder; think of pseudoephedrine.

